# Interplay between PI3K/AKT pathway and heart disorders

**DOI:** 10.1007/s11033-022-07468-0

**Published:** 2022-05-02

**Authors:** Soudeh Ghafouri-Fard, Ali Khanbabapour Sasi, Bashdar Mahmud Hussen, Hamed Shoorei, Afshan Siddiq, Mohammad Taheri, Seyed Abdulmajid Ayatollahi

**Affiliations:** 1 Department of Medical Genetics, School of Medicine, Shahid Beheshti University of Medical Sciences, Tehran, Iran; 2 Biochemistry Group, School of Medicine, Golestan University of Medical Science, Gorgan, Iran; 3 Department of Pharmacognosy, College of Pharmacy, Hawler Medical University, Kurdistan, Erbil, Iraq; 4 Center of Research and Strategic Studies, Lebanese French University, Kurdistan, Erbil, Iraq; 5 Department of Anatomical Sciences, Faculty of Medicine, Birjand University of Medical Sciences, Birjand, Iran; 6 Department of Pharmacology, Faculty of Pharmacy, University of Karachi, Karachi, Pakistan; 7 Institute of Human Genetics, Jena University Hospital, Jena, Germany; 8 Phytochemistry Research Center, Shahid Beheshti University of Medical Sciences, Tehran, Iran

**Keywords:** PI3K/AKT pathway, Myocardial infarction, Heart disease, Expression, Cardiac hypertrophy

## Abstract

The PI3K/AKT signaling has crucial role in the regulation of numerous physiological functions through activation of downstream effectors and modulation of cell cycle transition, growth and proliferation. This pathway participates in the pathogenesis of several human disorders such as heart diseases through regulation of size and survival of cardiomyocytes, angiogenic processes as well as inflammatory responses. Moreover, PI3K/AKT pathway participates in the process of myocardial injury induced by a number of substances such as H_2_O_2_, Mercury, lipopolysaccharides, adriamycin, doxorubicin and epirubicin. In this review, we describe the contribution of this pathway in the pathoetiology of myocardial ischemia/reperfusion injury and myocardial infarction, heart failure, cardiac hypertrophy, cardiomyopathy and toxins-induced cardiac injury.

## Introduction

The PI3K/AKT signaling has essential function in the regulation of numerous physiological processes through activation of downstream effectors which participate in the cell cycle transition and cell proliferation [1]. PI3K is lipid kinase that can phosphorylate the D3 hydroxyl group of the inositol ring of phosphoinositide lipids [2]. Based on their affinity for lipid substrates and their structure, PI3Ks can be classified into three main classes [3]. Different extracellular stimuli such as growth factors, cytokines and hormones can induce activity of PI3K. For instance, binding of EGF, PDGF and insulin-like growth factor [4, 5] to the RTK region can induce autophosphorylation of certain tyrosine residues in the cytoplasmic section leading to activation of PI3K. Moreover, activity of PI3K can be induced by G-protein coupled receptors [6].

AKT has three isoforms with the first two ones having ubiquitous expression and high levels of expression in the brain, heart and lung [7]. Growth factors and G-protein coupled receptors can stimulate PtdIns [3, 4] P2 and PtdIns [3–5] P3 to induce AKT recruitment to the plasma membrane, where it is phosphorylated at Thr308 and made active by PDK1 [8]. Subsequent phosphorylation of Ser473 residue is needed for full activity of AKT [9]. Following activation, AKT can phosphorylate a number of downstream targets, including GLUT, GSK-3, and mTOR [10]. PI3K/AKT pathway has imperative roles in the pathogenesis of several human disorders such as heart diseases through regulation of size and survival of cardiomyocytes, angiogenic processes as well as inflammatory responses [11]. In the current review, we describe the role of this pathway in the pathoetiology of myocardial ischemia/reperfusion (I/R) injury and myocardial infarction (MI), heart failure, cardiac hypertrophy, cardiomyopathy and toxins-induced cardiac injury. Cardiovascular disorders are constantly ranked as the foremost source of demise in the United States, surpassing all kinds of malignancies [12]. Coronary artery diseases have a prevalence of 7%. The incidence of heart failure ranges from 3.4 (per 1000 person years) for white women to 9.1 for black men [12].

## Myocardial ischemia/reperfusion (I/R) injury, myocardial infarction (MI) and heart failure (HF)

**Fig. 1:**
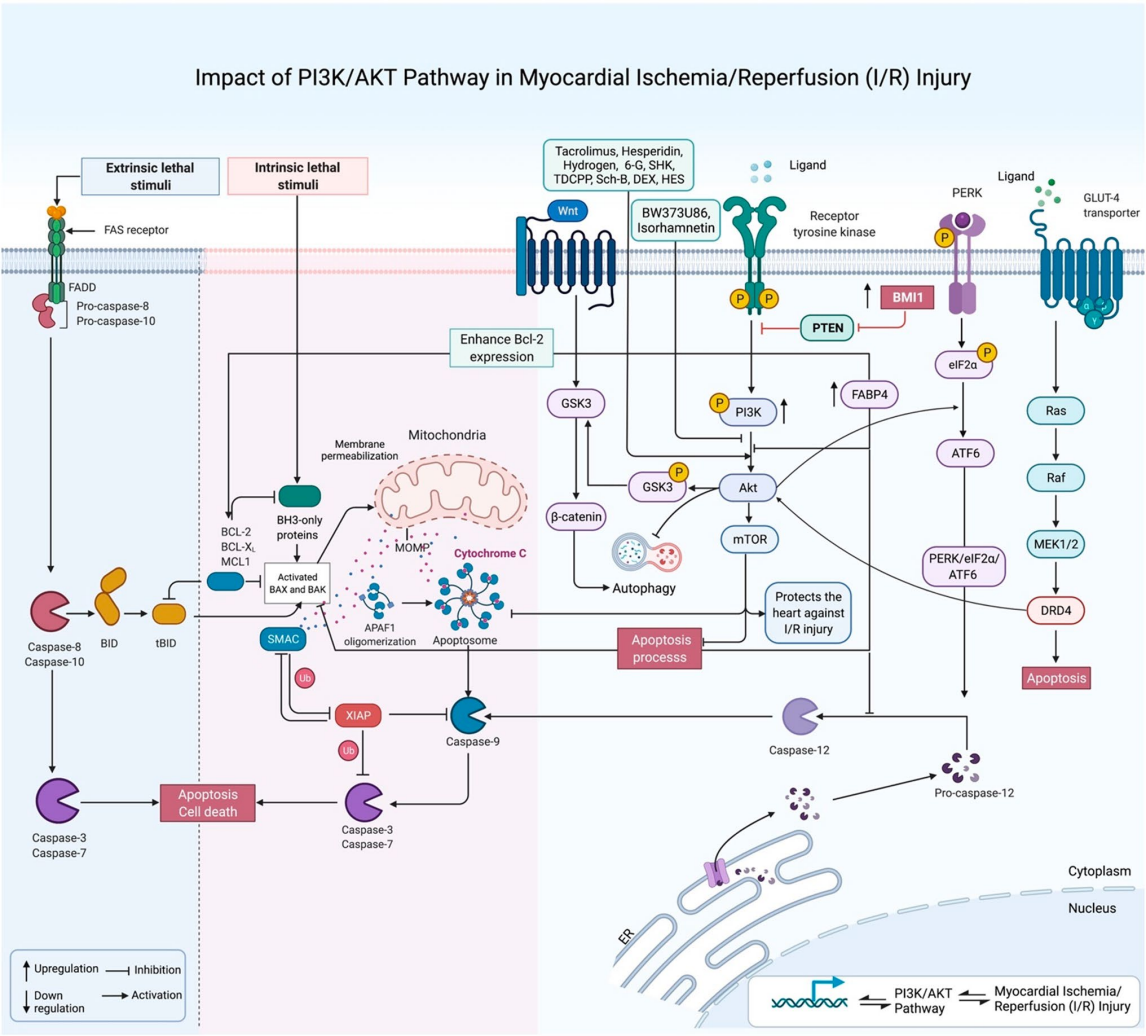
The PI3K/AKT pathway plays a critical role in the regulation of cardiomyocyte function, as well as the regulation of their growth and survival. This figure shows the role of PI3K/AKT-modulating pathway in the pathoetiology of myocardial ischemia/reperfusion (I/R) injury and myocardial infarction (MI), heart failure, cardiac hypertrophy, cardiomyopathy and toxins-induced cardiac injury

Myocardial I/R injury has been shown to be induced by endoplasmic reticulum stress and consequent apoptotic processes. Experiments in a cellular model of hypoxia reoxygenation have shown up-regulation of the fatty acid binding protein FABP4 injured cells. siRNA-mediated silencing of this gene has led to enhancement of cell viability and reduction of LDH levels following hypoxia reoxygenation process. Moreover, FABP4 silencing has decreased rate of apoptosis and activity of caspase-3 following this challenge. FABP4 down-regulation could also inhibit endoplasmic reticulum stress through reduction of p-PERK, GRP78, and ATF6 levels and block the endoplasmic reticulum stress-associated apoptotic pathway. The latter has been reflected in reduction of pro-apoptotic molecules p-JNK, CHOP, Bax, and caspase-12, along with enhancement of expression levels of Bcl-2. Most notably, FABP4 silencing has led to activation of the PI3K/AKT signaling. Cumulatively, FABP4 silencing has a protective effect against hypoxia reoxygenation damage through suppression of apoptosis through increasing activity of the PI3K/AKT pathway [13] (Fig. 1).

Another experiment in an animal model of isoproterenol-induced myocardial ischemic injury has shown that administration of the traditional Chinese medicine chishao along with terpene glycoside can decrease serum concentrations of CK and LDH, improve energy metabolism and relieve myocardial injury. Notably, this therapeutic regimen could increase levels of p-AKT and p-mTOR, while decreasing caspase-3 and Bax/Bcl-2 levels. These effects have been abolished following exposure with a PI3K inhibitor, indicating that the cardioprotective effects of chishao-terpene glycoside are exerted through induction of activity of PI3K/AKT/mTOR signaling [14].

Another combined in vitro and in vivo study has shown that urolithin A alleviates hypoxia/reoxygenation injury in myocardial cells and reduces size of MI and cell death in animals exposed to I/R. This agent could also enhance antioxidant aptitude of cardiomyocytes following mentioned challenge and reduce apoptosis of myocardial cells. Since these effects have been abrogated by a PI3K/AKT inhibitor, it has been revealed that urolithin A improves cardiac function following I/R injury probably via modulation of this pathway [15].

The cardioprotective triterpenoid Araloside C has been shown to suppress hypoxia-reoxygenation-induced apoptosis of cardiomyocytes, improve cell viability and attenuate the LDH leakage. Moreover, this agent could inhibit hypoxia-reoxygenation-induced endoplasmic reticulum stress through reduction of PERK/eIF2α and ATF6 activities and down-regulation of CHOP and caspase-12. These effects have been attributed to its impact on induction of HSP90 expression [16]. A mixed in vitro and in vivo study has shown that up-regulation of BMI1 promotes cardiac fibrosis, deteriorate cardiac function and enhances proliferation and migratory potential of fibroblasts. On the other hand, BMI1 silencing has attenuated cardiac fibrosis and stopped cardiac dysfunction. Moreover, up-regulation of BMI1 has decreased expression of PTEN, increased expression of PI3K, and enhanced phosphorylation of Akt and mTOR (Fig. 1). Notably, a PI3K/mTOR inhibitor could reverse the impact of BMI1 on cardiac fibroblasts. Taken together, BMI1 participates in the MI-associated cardiac fibrosis and dysfunction through influencing proliferation and migratory potential of cardiac fibroblasts at least partly via modulation of the PTEN/PI3K/AKT/mTOR pathway [17]. Table 1 shows the role of PI3K/AKT pathway in I/R injury, MI and HF.

**Table 1:** Impact of PI3K/AKT pathway in myocardial ischemia/reperfusion (I/R) injury

Drugs or supplements	Animal or human study & doses	Cell line	Dose	Targets/ main pathways	Conclusion	References
–	–	H9c2	–	FABP4, PERK, GRP78, ATF6α, JNK, Bcl-2, Bax, Caspase-12; *PI3K/AKT*	Silencing FABP4 via attenuating ER-mediated apoptosis by inducing the PI3K/AKT pathway could ameliorate H/R injury	[13]
CS & TG	SD Rats; 150 & 300 mg/kg, Daily, Orally, 7 consecutive days of pretreatment	H9c2	100 μg/mL	AMPK, Bcl-2, Bax, Caspase-3; *PI3K/AKT, mTOR*	CS-TG via inducing the PI3K/AKT/mTOR pathway could protect against isoproterenol-induced myocardial I/R injury	[14]
Urolithin-A	C57BL/6 mice; 1 mg/kg, I.P., pretreatment	–	–	Bcl-2, Bax, Caspase-3; *PI3K/AKT*	Urolithin-A via the PI3K/AKT pathway can amend myocardial I/R injury	[15]
Celastrol	SD Rats; 4 mg/kg, pretreatment	–	–	HMGB1, Bax, Bcl-2, LC3, Beclin-1; *PI3K/AKT*	Pretreatment with celastrol via the PI3K/AKT pathway through HMGB1 could reduce myocardial I/R injury	[18]
Nobiletin	SD Rats, 30 & 45 mg/kg, at the start of myocardial reperfusion	–	–	GRP78, CHOP, Cyt-c, Caspase-8/12; *PI3K/AKT*	Nobiletin by attenuating ER-associated apoptosis via regulating the PI3K/AKT pathway could ameliorate I/R injury	[19]
IGF-1	SD Rats; 1 or 5 mg/kg, injected via the caudal vein, before ischemia induction [10 min]	–	–	Caspase-9, Bcl-2; *PI3K/AKT*	IGF-1 via activating the PI3K/AKT pathway could act against I/R injury	[20]
T3	C57bl/6 mice; 2 µg/100 mg, I.P., 4 days before the experiment	NMVCs	20–80 ng/ml	Bax, Bcl-2, HO-1, Caspase-3/9, Nrf2; *PI3K/AKT*	Thyroid hormone via PI3K/AKT pathway could protect cardiomyocyte from H_2_O_2_-associated oxidative stress	[21]
Tacrolimus	SD Rats; 2 &100 mg/kg, I.P., 30 min prior to MIRI	–	–	Bcl-2, Bax, PPAR*γ,* Caspase-3; *PI3K/AKT*	Tacrolimus via activating the PPARγ/PI3K/AKT pathway could protect against I/R injury	[22]
Hesperidin	SD Rats; 200 mg/kg, Daily, for 3 days, pretreatment	–	–	LC3 II/I, Beclin-1, *PI3K/AKT, mTOR*	Hesperidin via activating the PI3K/AKT pathway can protect the heart against I/R injury by suppressing excessive autophagy	[23]
Hydrogen	SD Rats; 0.6 mmol/L in water	–	–	FoxO1, Bim, Caspase-3, *PI3K/AKT*	Hydrogen-rich water by activating the PI3K/AKT pathway could alleviate MIRI and inhibit cardiomyocytes apoptosis	[24]
BW373U86	SD Rats	Cardiac myocytes	5 mmol/L	LC3-II/IB, SQSTM1, p62; *PI3K/AKT, mTOR*	BW373U86 through suppression of the PI3K/AKT pathway and regulation of mTOR could upregulate autophagy to protect cardiomyocytes against H/R injury	[25]
6-G	SD Rats; 6 mg/kg, Pretreatment, Tail vein injection	–	–	Caspase-3, *PI3K/AKT*	6-G via inducing PI3K/AKT pathway could inhibit apoptosis to attenuate MIRI	[26]
SHK	–	H9c2	10, 20,and 40 μM	Bcl-2, Bax, caspase-3, Cyt-c; *PI3K/AKT*	SHK via inducing the PI3K/AKT pathway can protect H9C2 cardiomyocytes against H/R injury	[27]
Elabela	0.7 mg/kg, Tail vein injection, at 5 min of reperfusion	H9c2	5 nM	Cyt-c, caspase-3, Collagen-I/III, Bcl-2, Bax; *PI3K/AKT*	Elabela via PI3K/AKT pathway could alleviate myocardial I/R-induced apoptosis, fibrosis, and dysfunction of mitochondria	[28]
TDCPP	–	H9c2	0–50 μM	GSK-3β, Bcl-2, Bax, caspase-3, LC3-I/II; *PI3K/AKT*	TDCPP via activating the PI3K/AKT pathway could protect cardiomyocytes against H_2_O_2_-induced injury	[29]
Sch-B	SD rats; 60 mg/kg, gavage, daily, for 15 days	–	–	Bcl-2, Bax, caspase-3; *PI3K/AKT*	Sch-B via the PI3K/AKT pathway could protect against MIRI in rats	[30]
6-G	SD rats; 6 mg/kg, pretreatment 30 min before LAD ligation, via tail vein	–	–	TNF-α, IL-6, IL-1β, NLRP3, caspase-1; *PI3K/AKT*	6-G via the PI3K/AKT pathway could protect the heart by suppressing MIRI-induced inflammation	[31]
DEX	SD rats; 10 & 100 μg/kg, I.P., 30 min before the ischemia induction	–	–	Bax, Bcl-2, Bad, caspase-3; *PI3K/AKT*	Pretreatment with DEX via activating the PI3K/AKT pathway could protect against apoptosis in I/R injury	[32]
TBA	SD Rats; 0.5 & 1, 2 μg/ml 20 min before I/R	H9c2	3.125–100 μg/ml	CHOP, caspase-12, Bcl-2, Bax, ATF6, eIf2α, JNK; *PI3K/AKT*	TBA via the PI3K/AKT pathways by inhibiting ER-regulated apoptosis can protect against I/R injury	[33]
Troxerutin	SD Rats; 150 mg/kg, gavage, 4 weeks, before I/R	H9c2	0, 5, and 50 µM	Bax, Bcl-2, Caspase-3; *PI3K/AKT*	Troxerutin via the PI3K/AKT pathway can protect against I/R injury	[34]
DEX	SD Rats; 10 μg/kg, I.V., 5 min before reperfusion, and another injection to 120 min of reperfusion	–	–	GSK-3β, Bax, Bcl-2; *PI3K/AKT*	DEX postconditioning through activation of the PI3K/AKT pathway could increase the phosphorylation of GSK-3β and impede apoptosis and oxidative stress	[35]
Kaempferide	SD Rats; 0.1, 0.3, and 1 mg/kg, 30 min before I/R, then subjected to a 30 min LAD coronary artery ligation followed by a 2 h reperfusion	–	–	GSK-3β, Nrf-2, Caspase-3; *PI3K/AKT*	Kaempferide through induction of the PI3K/AKT pathway could protect against I/R injury	[36]
HES	SD Rats	NRCMs	6.25, 25, and 100 μM	Bcl-2, Bax, Caspase-3; *PI3K/AKT*	HES post-treatment via the activating PI3K/AKT pathway could prevent rat cardiomyocytes from H/R injury	[37]
–	SD Rats	H9c2	–	NEDD4-1, Bcl-2, Bax, Caspase-3; *PI3K/AKT*	NEDD4-1 via the PI3K/AKT pathway could protect against I/R-induced cardiomyocytes apoptosis	[38]
NGR1	–	H9c2	3.125–100 μg/ml	ERb/a, Caspase-3, Bcl-2, Bax; *PI3K/AKT*	NGR1 via the PI3K/AKT pathway could prevent H9c2 apoptosis against H/R	[39]
DRD4 agonist (PD168077)	SD Rats	AMCs, NRVMs	10^−5^ M	Caspase-3, GLUT4, Bcl-2, Bax; *PI3K/AKT*	DRD4 (dopamine receptor D4) in association with PI3K/AKT mediated glucose metabolism could mitigate myocardial I/R injury	[40]
HMGB1	SD Rats; 200 ng HMGB1 at 30 min before the I/R injury, I.V	–	–	VEGF; *PI3K/AKT*	HMGB1 via the PI3K/AKT pathway-mediated upregulation of VEGF expression could protect the heart against I/R injury	[41]
AS-IV	SD Rats; 20 & 50 mg/kg, Daily, 2 weeks, Gavage	HUVECs	10–160 μmol/L	PTEN, VEGF, Bcl-2, Bax; *PI3K/AKT*	AS-IV via regulating the PTEN/PI3K/AKT pathway could exert angiogenesis and cardioprotection after acute MI	[42]
DBE	C57BL/6 mice	–	–	JAK2, STAT3, VEGF, COX2, PPARγ, HIF-1α; *PI3K/AKT, mTOR*	DBE via PI3K/AKT/mTOR signaling could exert cardio-protection against injury in acute MI	[43]
–	C57BL/6 J mice	–	–	miR-23a-5p; *PI3K/AKT*	miR-23a-5p by inhibiting the PI3K/AKT pathway could induce MI by promoting cardiomyocytes apoptosis	[44]
–	SD Rats	–	–	SIRT1, PGC-1α, SOD-1/2, Collagen-I/III; *PI3K/AKT*	Postinfarction exercise training by biogenesis of mitochondria and SIRT1/PGC-1α/PI3K/AKT signaling could alleviate cardiac dysfunction	[45]
–	C57BL/6 mice	H9c2	–	GATA4, miR-221, PTEN, Caspase-3; *PI3K/AKT*	Overexpression of GATA4 via the miR-221-mediated targeting of the PTEN/PI3K/AKT pathway could enhance the antiapoptotic effect of exosomes secreted from cardiac fibroblasts	[46]
BP	SD Rats	ADSCs	7, and 20 μg/ml	STAT3, α-SMA; *PI3K/AKT*	Preconditioned adipose-derived stem cells via the PI3K/STAT3 pathway could ameliorate cardiac fibrosis through modulation of macrophage polarization	[47]
Leonurine	SD Rats; 15 & 30 mg/kg, Daily, Gavage, after the onset of MI for 28 days	–	–	GSK-3β, Bcl-2, Bax, Caspase-3; *PI3K/AKT*	Leonurine via the PI3K/AKT/GSK-3β pathway could protect cardiac function following acute MI	[48]
Ginsenoside Rg1	SD Rats; 10 mg/kg, Gavage, 60 min before ischemia	H9c2	0–200 μM	HIF-1α, Bax, Bcl-2, p62, Caspase-3/9, LC3-I/II, iNOS, Beclin-1; *PI3K/AKT, mTOR*	Ginsenoside Rg1 via the PI3K/AKT/mTOR pathway could protect cardiomyocytes from hypoxia-induced heart injury	[49]
Melatonin	C57BL/6 mice; 20 mg/kg, Daily, I.P	H9c2	–	Bcl-2, Bax, Caspase-3; *PI3K/AKT*	Melatonin through the PI3K/AKT pathway could alleviate hypoxia-induced cardiac apoptosis	[50]
rhBNP	–	H9c2	200, 600, and 900 nmol/L	lncRNA EGOT, Cyclin-D1, LC3-II/I, Beclin-1, Bcl-2, Bax, p62, Caspase-3/9; *PI3K/AKT, mTOR*	rhBNP via lncRNA EGOT could regulate PI3K/AKT/mTOR pathway to reduce hypoxia-induced heart injury	[51]
Araloside-C	SD Rats; 2.5 mg/kg/day; for 4 weeks	–	–	Bax, Bcl-2, Cyt-c, Caspase-3; *PI3K/AKT*	Araloside-C by regulating the PI3K/AKT could prevent myocardial cell apoptosis to relieve HF	[16]
BMI1	C57BL/6 mice; 5 × 10^6^ transducing units of BMI1 RNA-interfering lentivirus for 5 points	–	–	PTEN, BMI1; *PI3K/AKT*, *mTOR*	BMI1 via the PTEN and PI3K/AKT/mTOR pathways could promote cardiac fibrosis in ischemia-induced HF	[17]
KF	SD Rats; 10 and 20 mg/kg, 42 consecutive days, orally	–	–	Nrf-2, NF-κβ, GSK-3β; *ERK/MAPK, PI3K/AKT*	KF via reducing the alterations in pathways such as the PI3K/AKT/GSK-3β could inhibit oxidative stress, inflammation, and apoptosis	[52]
–	C57 mice	H9c2	–	miR-181c, TNF-α, Bcl-2, Caspase-3, Bax; *PI3K/AKT*	miR-181c through PI3K/AKT signaling pathway could protect cardiomyocytes injury by preventing cell apoptosis	[53]
QSKL	SD Rats; 2.33 g/kg, Daily, for 28 days, dissolved in water	H9c2	400, 600, and 800 μg/ml	Caspase-3, Bcl-2, Bax, P53, PTEN; *PI3K/AKT*	QSKL via the PI3K/AKT-p53 pathway can protect against myocardial apoptosis in HF	[54]
FA	C57BL/6 mice; 25–100 mg/kg, Gavage, Daily, for 7 days	Cardiomyocytes	0–160 μM	α-SMA, TGF-β1, Collagen-I/III, ERK1/2, SMAD2/3; *PI3K/AKT*	FA via the TGF-β1/SMADs and PI3K/AKT pathways could protect HF induced by isoproterenol	[55]
–	SD Rats; 48 pairs of healthy control and patients with CHD	PBMCs	–	TET2, miR-126, E2F3; *PI3K/AKT*	TET2 by promoting miR-126 and suppression of the E2F3/PI3K/AKT axis could expedite CHD	[56]
AGIV	SD Rats	RAECs	10^–2^–10^–4^ mM	eNOS; *PI3K/AKT*	AGIV by regulating the PI3K/AKT/eNOS pathway could improve vasodilatation function in RAECs	[57]
–	BALB/c mice	VSMCs	–	PTEN, Bax, Bcl-2, Caspase-3; *PI3K/AKT*	Ectopic expression of PTEN via the PTEN/PI3K/AKT pathway could promote apoptosis in VSMCs	[58]
Anthocyanin	SD Rats; 250 mg/kg, Daily, for 4 weeks, Gavage	–	–	IGF-1R, Caspase-3/8/9, Bad, Bak, Cyt-c, Bcl-2, Bak; *PI3K/AKT*	Anthocyanin suppresses cellular apoptosis and cardiac dysfunction in STZ-induced diabetic rats through activation of IGFI-R/PI3K/AKT	[59]

## Cardiac hypertrophy

Guan et al. have exposed male rats were to CIH and/or resveratrol to examine the cardioprotective effect of resveratrol and clarify the underlying mechanism. They have reported that CIH increases heart weight/body weight ratio and left ventricle weight/body weight ratio and induces left ventricular remodelling. Moreover, CIH has increased left ventricular posterior wall thickness, ejection fraction and fractional shortening, and increased apoptosis index and expression of oxidative markers. Notably, resveratrol could improve cardiac function and alleviate cardiac hypertrophy, oxidative stress, and apoptosis in CIH-treated rats. Mechanistically, resveratrol-induced activation of autophagy has been shown to be exerted through PI3K/AKT pathway-associated suppression of mTOR [60].

The traditional herbal medicine Qingda granule has also been demonstrated to protect against Ang II-induced cardiac hypertrophy through modulation of PI3K/AKT pathway. This agent could attenuate the Ang II-induce rise in blood pressure and decrease left ventricle ejection fraction and fractional shortening. Besides, Qingda granule could alleviate the increase in the heart weight/tibia length ratio, cardiac damage, hypertrophy, and apoptosis. In vitro investigations has verified the impact of Qingda granule in amelioration of the Ang-II-induced enhancement of cell surface area and quantities of apoptotic cells, increase in the expression of ANP and BNP, and activity of caspases-9 and -3. Notably, Qingda granule could partially amen accretion of ROS, mitochondrial membrane depolarization, cytochrome C release, over-expression of Bax, and reduction of p-PI3K, p-AKT, and Bcl-2 [61].

Another Chinese herbal medicine, namely Isorhamnetin has been found to guard against cardiac hypertrophy through modulation of this pathway [62]. Table 2 shows the role of PI3K/AKT Pathway in cardiac hypertrophy.

**Table 2:** PI3K/AKT pathway in cardiac hypertrophy

Drugs or supplements	Animal or human study & doses	Cell line	Dose	Targets/ main pathways	Conclusion	References
RESV	SD Rats; 30 mg/kg, Daily, 5 weeks, Gavage	–	–	LC3-II/I, Beclin-1, p62, Bcl-2, Bax; *PI3K/AKT, mTOR*	RESV by targeting the PI3K/AKT/mTOR pathway can defend chronic intermittent hypoxia-associated cardiac hypertrophy	[60]
QDG	C57BL/6 mice; 1.145 g/kg/day, Orally, for 2 weeks	H9c2	0.05 mg/mL	ANP, BNP, Cyt-c, Bax, Bcl-2; *PI3K/AKT*	QDG by activating the PI3K/AKT pathway could reduce Ang II-induced hypertension, cardiac hypertrophy, and apoptosis	[61]
Isorhamnetin	SD Rats; 100 mg/kg, Daily, after Aortic banding (AB) surgery, for 8 weeks, with vehicle	NRCMs	5–100 μM	GSK-3β, eIF-4E, P70S6K; *PI3K/AKT, mTOR*	Isorhamnetin through blocking PI3K/AKT pathway could protect against cardiac hypertrophy	[62]

## Diabetic cardiomyopathy

Carvacrol as a natural cymene-derived monoterpene has been found to reduce blood glucose levels and suppress diabetic-induced cardiac remodeling in animal models. These effects have been accompanied by down-regulation of Nppa and Myh7 mRNAs reduction of cardiac fibrosis. Notably, carvacrol can reestablish PI3K/AKT signaling, which was compromised in diabetic mice. This substance has enhanced phosphorylation of PI3K, PDK1, AKT, and AS160 and decreased phosphorylation of PTEN in these animals. Finally, Carvacrol has been shown to enhance membrane translocation of GLUT4. Cumulatively, the protective effect of Carvacrol against diabetic cardiomyopathy is exerted through reestablishing PI3K/AKT signaling-facilitated translocation of GLUT4 to the cell membrane [63].

Nicorandil has been shown to exert anti-apoptotic roles in diabetic cardiomyopathy. This drug could enhance serum level of NO and cardiac level of eNOS in the diabetic animals, amend cardiac dysfunction and decrease apoptosis rate. These effects have been blocked by administration of 5-HD, a substance that inhibits phosphorylation of PI3K, Akt, eNOS, and mTOR. Thus, the anti-apoptotic effect of nicorandil in diabetic cardiomyopathy is exerted through modulation of PI3K/Akt pathway [64]. Similarly, another experiment in a rat model of diabetic cardiomyopathy has shown that resveratrol amends heart dysfunction through suppression of apoptosis via the PI3K/AKT/FoxO3a pathway [65]. Table 3 shows the role of PI3K/AKT pathway in diabetic cardiomyopathy.

**Table 3:** Role of PI3K/AKT pathway in diabetic cardiomyopathy (DCM)

Drugs or supplements	Animal or human study & doses	Cell line	Dose	Targets/ main pathways	Conclusion	References
CAR	C57BL/6 J mice; 10 & 20 mg/kg, daily, for 6 weeks, I.P	–	–	p85, PDK1, PTEN, GLUT4, AS160; *PI3K/AKT*	CAR by modulating the PI3K/AKT/GLUT4 pathway could attenuate DCM	[63]
Nicorandil	SD Rats; 7.5 and 15 mg/kg, daily, for 4 weeks, drinking water	H9c2	10, 50, and 100 μmol	MMP2/9, Bcl-2, Bax, collagen-I/III, caspase-3, eNOS; *PI3K/AKT*	Nicorandil via the PI3K/AKT pathway can alleviate apoptosis in DCM	[64]
RESV	SD Rats; 5 & 50 mg/kg, daily, gavage, for 8 weeks	Neonatal rat ventricular myocytes	10 µM	Bcl-2, Bax, FoxO3a; *PI3K/AKT*	RESV via the PI3K/AKT/FoxO3a pathway could ameliorate cardiac dysfunction by inhibiting apoptosis in a rat model of DCM	[65]

## Other conditions

An in vitro study in H_2_O_2_-induced H9c2 cells has shown down-regulation of miR-129-5p. Moreover, this treatment has resulted in reduction of cell viability and induction of cell autophagy. Forced up-regulation of miR-129-5p could inhibit H_2_O_2_-induced cell injury. Besides, ATG14 has been fund tp be a target of miR-129-5p. miR-129-5p overexpression could also activate phosphorylation of PI3K/AKT/mTOR pathway resulting in reduction of the autophagy and apoptosis in H_2_O_2_ exposed cells. Taken together, miR-129-5p has a protecting role against H_2_O_2_-induced autophagy and apoptosis through decreasing levels of ATG14 via activating of PI3K/AKT/mTOR pathway [66].

An in vivo study has demonstrated that Luteolin could ameliorate HgCl_2_-induced cardiac damage via mediating the PI3K/AKT/Nrf-2 pathway [67].

Salidroside, the glucoside of tyrosol derived in the plant Rhodiola rosea has been found to have protective effect against lipopolysaccharide (LPS)-induced myocardial injury through modulation of PI3K/AKT pathway. Mechanistically, this substance can reduce iNOS, COX-2 and NF-κB levels and decrease activity of PI3K/Akt/mTOR pathway [68]. Moreover, the plant-derived flavone Apigenin (4′,5,7-trihydroxyflavone) has been shown to attenuate adriamycin-induced cardiomyocyte apoptosis via modulation of PI3K/AKT/mTOR pathway [69]. Table 4 shows the role of PI3K/AKT pathway in different cardiac disorders, particularly toxin-related conditions.

**Table 4:** Role of PI3K/AKT pathway in other heart diseases

Diseases	Drugs or supplements	Animal or human study & doses	Cell line	Dose	Targets/ main pathways	Conclusion	References
Myocardial injury by H_2_O_2_	–	–	ATCC, CRL-1446	–	miR-129-5p, ATG14, p62, Beclin-1, LC3II, Bcl-2, Bax, Caspase-3; *PI3K/AKT, mTOR*	miR-129-5p via the PI3K/AKT/mTOR pathway could inhibit apoptosis and autophagy in H9c2 cells treated with H_2_O_2_	[66]
Myocardial injury by mercury	Luteolin	SD rats; 80 mg/kg, daily, gavage, in the last 14 days	–	–	Nrf-2, HO-1, NQO1, NF-κB, TNF-α, P53, Bax, Caspase-3, Bcl-2; *PI3K/AKT*	Luteolin via mediating the PI3K/AKT/Nrf-2 pathway could ameliorate HgCl_2_-induced cardiac damage	[67]
Myocardial injury by LPS	Sal	SD rats; 20 & 40 mg/kg, gavage, daily, for 3 days	H9c2	10–160 μM	iNOS, COX-2, NF-κB; *PI3K/AKT, mTOR*	Sal by reducing ROS-mediated PI3K/AKT/mTOR pathway activity could suppress LPS-induced myocardial injury	[68]
Myocardial injury by adriamycin	API	Kunming mice; 125 & 250 mg/kg, I.P., at an interval of 48 h, for 17 days	–	–	Bcl-2, Bax, LC3BI/II, Beclin-1; *PI3K/AKT*,* mTOR*	API via the PI3K/AKT/mTOR signaling can attenuate adriamycin-induced cardiomyocyte apoptosis	[69]
Myocardial injury by Doxorubicin	CUR	Kunming mice; 50, 100, 200, and 400 mg/kg, gavage, daily, for 17 days	H9c2	10 μM	Bcl-2, Bax, Caspase-1, IL-1β, NLRP3, LC3-II/I, Beclin-1; *PI3K/AKT, mTOR*	CUR via the PI3K/AKT/mTOR-dependent manner could suppress doxorubicin-induced cardiomyocytes pyroptosis	[70]
Myocardial injury by epirubicin	Paeonol	BALB/c mice; 50 mg/kg, daily, gavage, for 6 days	H9c2, LH-1	100 μM	miR-1, Bcl-2, Bax, Caspase-3, TNF-α, LC3-II/I, Beclin1, Atg5, NF-κB; *PI3K/AKT, mTOR*	Phenol by suppressing the PI3K/AKT/mTOR and NF-kB pathways could ameliorate MI by increasing miR-1 expression	[71]
Tert-butyl hydroperoxide (TBHP)-induced heart injury	3,5-diCQA	–	H9c2	5–20 μM	Caspase-3, PTEN; *PI3K/AKT*	3,5-diCQA via activating the PI3K/AKT pathway could protect H9c2 cells against oxidative stress-induced apoptosis	[72]
Coronary microembolization (CME)	–	SD rats	–	–	miR-486-5p, PTEN, Caspase-3; *PI3K/AKT*	miR-486-5p via targeting PTEN by activating the PI3K/AKT pathway could protect against CME-induced cardiomyocyte apoptosis	[73]
Cardiac insulin resistance	–	SD Rats; 300 & 600 mg/kg, for 7 days, Gavage	–	–	*PI3K/AKT*	Caloric restriction by activating the PI3K/AKT pathway could attenuate aging-induced cardiac insulin resistance	[74]
Myocarditis	Lipoxin-A4	BALB/c mice; 10–50 μg/kg, Daily, I.P., for 3 weeks	–	–	IKKα/β; PI3K/AKT	Lipoxin-A4 by regulating NF-κB and PI3K/AKT pathway could mitigate experimental autoimmune myocarditis in mice	[75]
Hypertension	GABA tea	Rats; 100 and 300 mg/kg, daily, for 12 weeks	–	–	IGF1, bad, Bcl-2, bak, caspase-3/9; *PI3K/AKT*	GABA tea by enhancing PI3K/AKT-mediated activity and suppressing bax/bak could attenuate cardiac apoptosis	[76]
Hypertension	Fisetin	Rats; 10 mg/kg, twice a week, for 6 weeks, Orally	H9c2	50 μM	TNF-α, Caspase-3, Bax, Bcl-2, IGFIR; *PI3K/AKT*	Fisetin through induction of IGF-IR/PI3K/AKT signaling could act against angiotensin II-induced apoptosis	[77]
Physiological cardiac growth	Epicatechin	CD-1 mice; 1 mg/kg, twice a day, for 2 weeks, gavage	–	–	Collagen-III, β-MHC, p70S6K, *PI3K/AKT, mTOR*	Epi by activation of the PI3K/AKT pathway can induce physiological cardiac growth in healthy animals	[78]
Hyperthyroidism	Hydrogen sulfide (H_2_S)	SD Rats; 100 μmol/kg, I.P., daily, for weeks	–	–	miR-21, miR-34a, miR-214, miR-221, MMP-11/12/14/17, ATG5/7/16L1, beclin-1, LC3A; *PI3K/AKT*	H_2_S via the PI3K/AKT pathway could ameliorate rat myocardial fibrosis induced by thyroxin	[79]

## Discussion

PI3K/AKT pathway is involved in the regulation of fundamental cellular processes, including migration of cells, translational response, and survival of cells. Thus, it can modulate cellular metabolism, vascular homeostasis and thrombogenic processes [80]. Accordingly, PI3K/AKT pathway has central roles in the regulation of function of cardiomyocytes and their size and survival. Moreover, this pathway can regulate activity of immune cells. Thus, it is not surprising that this pathway participate in the pathoetiology of myocardial I/R injury and MI, heart failure, cardiac hypertrophy, cardiomyopathy and toxins-induced cardiac injury. Most conducted researches in this field have assessed the impact of this pathway in the pathoetiology of myocardial I/R injury and MI revealing several targets for modulation of the effects of PI3K/AKT pathway.

Notably, PI3K/AKT pathway has an essential role in cardiac fibrosis. Alterations in the cardiomyocytes during the pathogenic processes in cardiac fibroblasts, abnormal proportion of collagen I/III, and the disproportionate synthesis and deposition of extracellular matrix are affected by this pathway. This process is a shared pathological alteration occurring in many cardiac disorders such as ischemic heart disease, hypertension, and heart failure. The role of PI3K/AKT signaling in these processes pathway is exerted through regulation of cell survival, apoptosis, growth and contraction of cardiac cells. Moreover, this pathway can also modulate expression of mTOR, GSK-3, FoxO1/3, and NOS in this process [81]. Preliminary results of in vitro and in vivo studies have shown dual inhibition of PI3K/Akt and mTOR pathways using BEZ235 can attenuate process of fibrosis [82]. Future studies are needed to assess the effect of different inhibitors of these pathways on cardiac fibrosis.

Moreover, PI3K/AKT has a fundamental role in the pathological processes leading to atherosclerosis initiating from formation of atherosclerotic plaques their rupture. The PI3Kγ isoform of PI3K which is over-expressed in the hematopoietic cells has a particular role in induction of inflammation during atherosclerosis [83]. A number of atherogenic stimuli such as IFNγ, TGFβ, and TNF-a can also activate PI3K/AKT signaling [83]. Development of atherosclerosis can also be affected by PI3K/AKT pathway through modulation of migration of vascular smooth muscle cells, adhesion of platelets, and expression of inflammatory molecules [84].

The cardioprotective effects of several traditional medicines have been shown to be exerted through modulation of activity of this signaling pathway. For instance, resveratrol, Qingda granule and Isorhamnetin have been shown to protect against cardiac hypertrophy through modulation of PI3K/AKT pathway. Urolithin-A, Celastrol, Nobiletin, Tacrolimu, Hesperidin, Elabela, Troxerutin, Kaempferide, Leonurine, Ginsenoside Rg1, Melatonin, Araloside-C and Anthocyanin are examples of substances that protect against MI-induced I/R injury or heart failure through modulation of PI3K/AKT pathway. A number of transcription factors and miRNAs such as miR-23a-5p, miR-221, miR-126, miR-129-5p, miR-1, miR-486-5p have also been found to be implicated in the pathogenesis of heart disease through modulation of this pathway. These effects have been verified through experiments in cellular and animal models of heart injury. However, clinical studies are lacking in this field.

Most notably, PI3K/AKT pathway is regulated by several non-coding genes including miRNAs, long non-coding genes and circular RNAs [85]. This finding represents the complexity of regulation of PI3K/AKT pathway and indicates that any targeted therapy against this pathway should consider the effects of these transcripts on the activity of this pathway to yield the highest effectiveness with the lowest side effects.

## Conclusion

PI3K/AKT pathway represents a candidate for design of effective drugs for treatment of heart disorders and appropriate substances for prevention of these disorders. Based on the importance of PI3K/AKT pathway in the pathogenesis of heart diseases and abundance of therapeutic substances that modulate activity of this pathway, further researches in this field can facilitate discovery of novel modalities for treatment of heart disorders. Further studies are needed to find specific markers for identification of response of patients to PI3K/AKT-modulating agents used for treatment of heart disorders.

## Data Availability

Data sharing not applicable to this article as no datasets were generated or analysed during the current study.
